# Maslinic acid improves mitochondrial function and inhibits oxidative stress and autophagy in human gastric smooth muscle cells

**DOI:** 10.1515/biol-2022-1036

**Published:** 2025-01-27

**Authors:** Xiaoying Zheng, Shuning Zhang, Qiaobin Chen

**Affiliations:** Department of Pediatrics, Fujian Provincial Hospital, Shengli Clinical Medical College of Fujian Medical University, No. 134 Dong Street, Fuzhou, Fujian, 350001, China; Major of Pediatrics, Fujian Medical University, Fuzhou, Fujian, 350001, China

**Keywords:** autophagy, functional dyspepsia, maslinic acid, mitochondrial function, oxidative stress, the AMPK/SIRT1 pathway

## Abstract

Functional dyspepsia (FD) is a chronic disease that occurs in the gastroduodenal region and significantly impacts human health. Maslinic acid (MA), a pentacyclic triterpene acid, is the primary bioactive ingredient in Chinese medicinal herbs such as hawthorn, which exhibits beneficial impacts on the regulation of various disease progressions. However, the specific functions and associated pathways of MA in FD progression remain unclear and require further investigation. In this work, it was demonstrated that MA enhanced the cell viability of human gastric smooth muscle cells (HGSMCs). In addition, the mitochondrial dysfunctions induced by carbonyl cyanide 3-chlorophenylhydrazone (CCCP) were rescued after MA treatment. Furthermore, autophagy was increased following CCCP treatment, but this phenomenon was counteracted after MA treatment. The oxidative stress, elevated after CCCP treatment, was alleviated following MA addition. Finally, the AMPK/SIRT1 pathway was suppressed after CCCP stimulation but was re-activated after MA treatment. In conclusion, it was uncovered that MA accelerated HGSMC viability and improved mitochondrial function, inhibited autophagy, alleviated oxidative stress, and stimulated the AMPK/SIRT1 pathway. This discovery may offer new insight into the therapeutic effects of MA in FD progression.

## Graphical abstract

**Figure:**
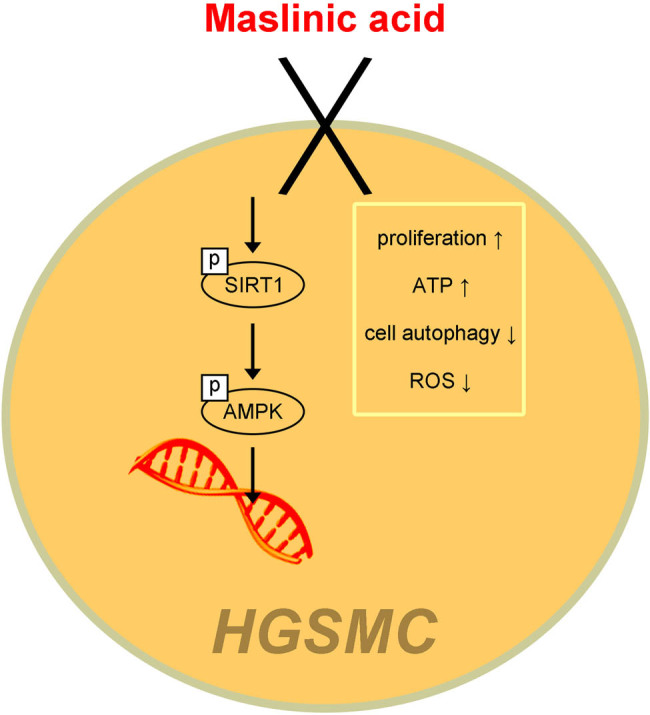

## 1 Introduction

Four types of diseases such as functional dyspepsia (FD), irritable bowel syndrome (IBS), functional abdominal pain (FAP), and abdominal migraine (AM) have been confirmed to be associated with adolescents’ functional gastrointestinal diseases [1,2]. FD refers to a chronic disease that occurs in the gastroduodenal region, which can cause early satiety during normal meals, postprandial satiety without any organic disease, and pain or a burning sensation in the upper abdomen [3,4]. Currently, no effective treatment has been identified for FD because of the complex etiology and pathophysiology of FD. As a result, the search for novel drugs remains a promising approach for the treatment of FD.

Maslinic acid (MA) is a pentacyclic triterpene acid and the primary bioactive component extracted from Chinese medicinal herbs (such as hawthorn, olive, and jujube) [5,6]. MA possesses antibacterial, antiviral, antioxidant, and anticancer activities and has attracted significant attention from the medical community. For instance, MA can inhibit cell apoptosis and necrosis, preserve the glomerulus, and thus exhibit a therapeutic effect on cisplatin-triggered nephrotoxicity in rats [7]. Additionally, MA attenuates inflammatory response, maintains the blood–milk barrier, and modulates intestinal flora, thereby relieving LPS-stimulated mastitis [8]. Furthermore, MA accelerates mitochondrial apoptosis and reduces HIF-1α expression in lung cancer [9]. Importantly, MA demonstrates gastroprotective activity through various mechanisms, which is beneficial for the mucus barrier [10]. Besides, an MA-enriched diet can affect transcriptomic and metabolomic reprogramming in Apc (Min/+) mice to alleviate intestinal tumorigenesis [11]. However, the detailed functions and associated pathways of MA in FD progression remain unclear.

In this work, it is purposed to explore the impacts of MA on mitochondrial function, autophagy, and oxidative stress. Our findings first revealed that MA enhanced human gastric smooth muscle cell (HGSMC) viability and improved mitochondrial function, inhibited autophagy, alleviated oxidative stress, and stimulated the AMPK/SIRT1 pathway. This study suggests that MA might be a promising drug for ameliorating FD progression.

## 2 Materials and methods

### 2.1 Cell lines, culture, and treatment

The HGSMCs were purchased from Shanghai Yaji Biotechnology Company (Shanghai, China). HGSMCs were incubated with the primary smooth muscle cell culture system (Icell, PriMed-iCell-004) containing 5% fetal bovine serum (FBS, Gibco Laboratories, USA) and penicillin/streptomycin antibiotic solution. HGSMCs were incubated at 37°C with 5% CO_2_ in Dulbecco’s modified Eagle’s medium (Gibco, New York, NY, USA) supplemented with 10% FBS. The culture media were changed every 48 h. HGSMCs from passages 4 to 6 were used in subsequent experiments.

Carbonyl cyanide 3-chlorophenylhydrazone (CCCP, 10 μM, Absin, Shanghai, China) was used for treating HGSMCs for 4 h to mimic the FD cell model [12]. MA (Shanghai Huicheng Biotechnology Company, Shanghai, China) with different concentrations (0, 10, 20, 40, 80, and 160 μM) was utilized for treating HGSMCs for 24 h [13].

Groups were divided into control (no treatment), CCCP, CCCP+MA 20 μM, CCCP+MA 40 μM, and CCCP+MA 80 μM group.

### 2.2 CCK-8 assay

The HGSMCs (1,000 cells/well) were seeded into the 96-well plate for 24 h. In each well, CCK-8 solution (10 μL, Dojindo Laboratories, Kumamoto, Japan) was added and incubated for 2 h. Finally, using the spectrophotometer (Thermo Fisher Scientific, MA, USA), cell viability was measured.

### 2.3 Detection of adenosine triphosphate (ATP)

The ATP level was determined by the ATP commercial kit (No. A095, Jiancheng Bioengineering Institute, Nanjing, China). Briefly, HGSMCs were re-suspended in ddH2O (500 μL). After homogenization, the mixture was centrifuged to collect the supernatant. The optical density value (636 nm) was confirmed by a spectrophotometer (Thermo Fisher Scientific, MA, USA).

### 2.4 JC-1 staining

Mitochondrial membrane potential (MMP) level was evaluated through JC-1 staining. The HGSMCs in different groups were treated with JC-1 solution (0.5 mL, Yeasen, Shanghai, China) for 15 min. Next, HGSMCs were centrifuged and then resuspended in a buffer solution. After washing, images were captured using a fluorescent microscope (Olympus, Tokyo, Japan).

### 2.5 Western blot

Proteins (extracted from HGSMCs) were separated under sodium dodecyl sulfate-polyacrylamide gel electrophoresis (10%). Next, proteins were transferred to polyvinylidene difluoride (PVDF, Beyotime, Shanghai, China) membranes. After blocking, the membranes were incubated with primary antibodies for 12 h and followed by secondary antibodies (1:2,000; ab7090; goat anti-rabbit IgG H&L [HRP]) for 2 h. Finally, using the chemiluminescence detection kit (Thermo Fisher Scientific, Inc.), the protein expressions were measured.

The primary antibodies used were as follows: LC3B (autophagy marker, 1:2,000; ab192890; Rabbit; Abcam, Shanghai, China), P62 (1:10,000; ab109012; Rabbit), p-SIRT1 (1:1,000; ab76039; Rabbit), SIRT1 (0.5 µg/mL; ab110304; Mouse), p-AMPK (1:1,000; ab92701; Rabbit), AMPK (1:1,000; ab32047; Rabbit), and GAPDH (1:500; ab8245; Mouse).

### 2.6 Immunofluorescence (IF) assay (for autophagy evaluation)

The LC3B fluorescence intensity was measured for autophagy evaluation. HGSMCs were seeded in the 24-well plates for 24 h. Next, cells were fixed with 4% paraformaldehyde for 20 min and permeabilized with 0.25% Triton X-100 for 10 min. After blocking, the coverslips were incubated with a primary antibody against LC3B (1 µg/mL; ab192890; Rabbit; Abcam, Shanghai, China), then followed by the fluorescent secondary antibody. For nuclear staining, 4′,6-diamidino-2-phenylindole dihydrochloride was used. Images were captured under one fluorescent microscope (Olympus, Tokyo, Japan).

### 2.7 Detection of ROS

The reactive oxygen species (ROS, E004-1-1, Nanjing Jiancheng Technology Co., Ltd., Nanjing, China) was used to evaluate the ROS fluorescence intensity. HGSMCs were incubated with dichloro-dihydro-fluorescein diacetate for 20 min. After washing with phosphate-buffered saline, the ROS fluorescence intensity was evaluated under the fluorescence microscope (Olympus, Tokyo, Japan).

### 2.8 Detection of malondialdehyde (MDA), superoxide dismutase (SOD), and glutathione peroxidase (GSH-Px) (oxidative stress)

MDA (ab118970, Abcam, Shanghai, China), SOD (ab65354), and GSH-Px (ab65322) commercial kits were adopted to evaluate the levels of MDA, GSH, and SOD in the supernatant.

### 2.9 Statistical analysis

All results were expressed as mean ± standard deviation (SD) with three times repetitions. Statistical analysis was performed using GraphPad Prism Software 9 (GraphPad Software, USA). The comparisons between the control, CCCP, CCCP+MA 20 μM, CCCP+MA 40 μM, and CCCP+MA 80 μM groups were conducted using the one-way ANOVA with Tukey’s post hoc test. The *p-*value of <0.05 was considered statistically significant.

## 3 Results

### 3.1 MA accelerated cell viability of HGSMCs

**Figure 1:**
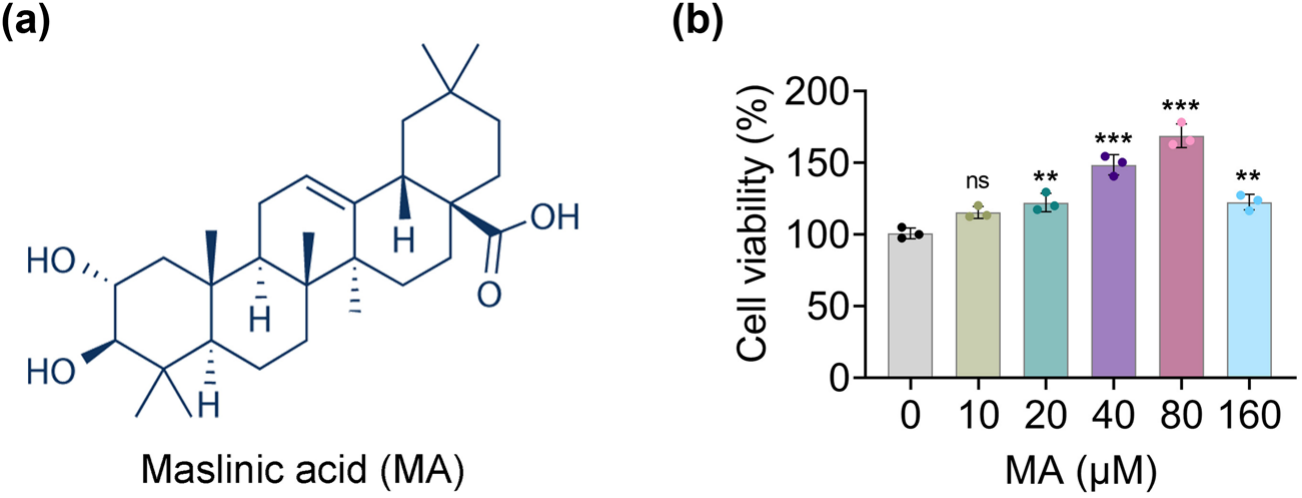
MA accelerated cell viability of HGSMCs. (a) The chemical structure of MA. (b) The cell viability of MA treatment (0, 10, 20, 40, 80, and 160 μM) was confirmed through the CCK-8 assay. ***p < 0.001 vs MA (0 μM).

The chemical structure of MA is displayed in Figure 1a. Next, it was demonstrated that cell viability was gradually increased after MA treatment (20, 40, 80, and 160 μM), and 20, 40, and 80 μM MA treatments were chosen for further experiments (Figure 1b). In summary, MA accelerated the cell viability of HGSMCs.

### 3.2 MA ameliorated mitochondrial function in HGSMCs

**Figure 2:**
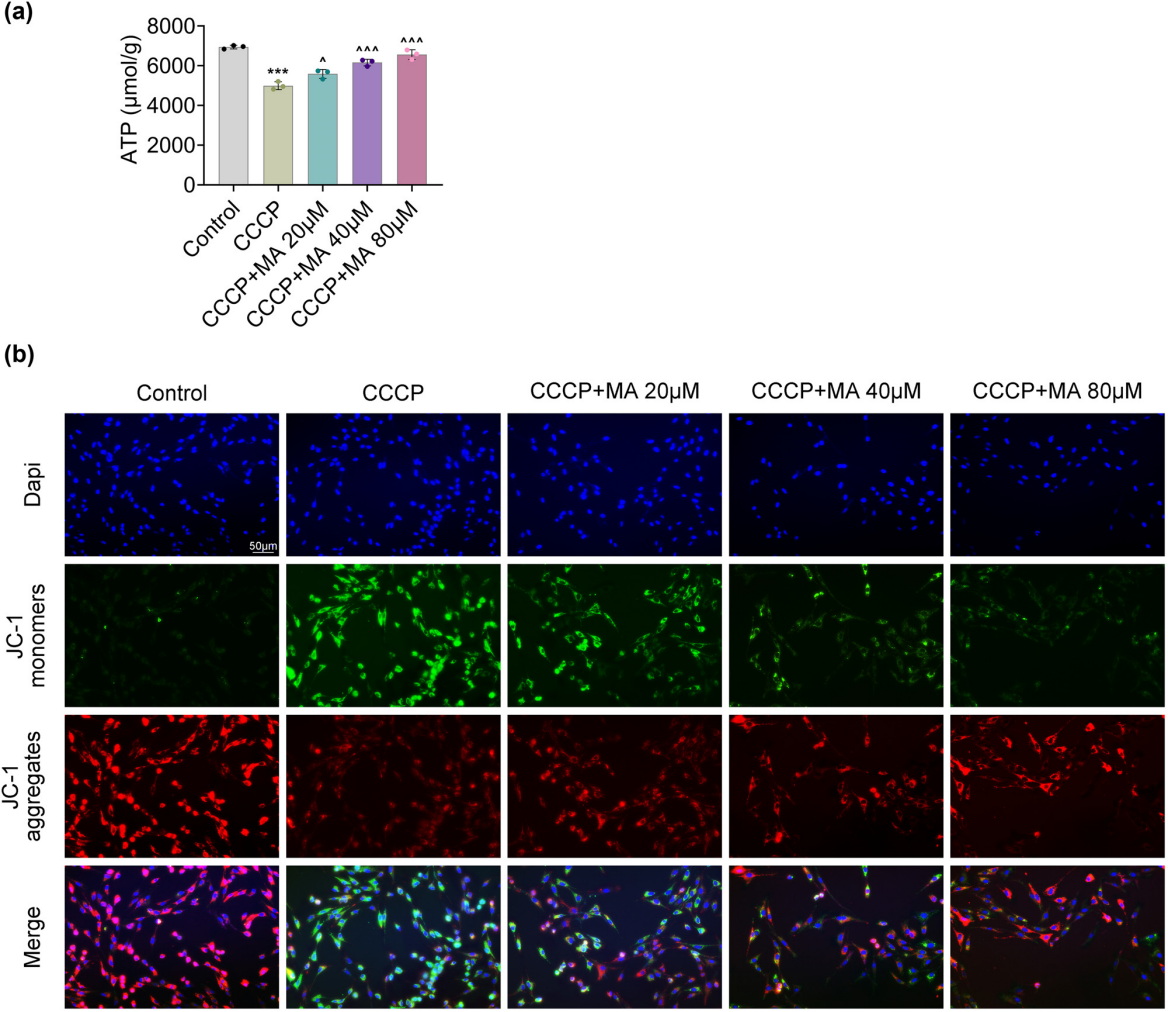
MA ameliorated mitochondrial function in HGSMCs. Groups were separated into the control, CCCP, CCCP+MA 20 μM, CCCP+MA 40 μM, and CCCP+MA 80 μM group. (a) The ATP level was confirmed through the ATP commercial kit. (b) The MMP level was evaluated through JC-1 staining. ***p < 0.001 vs the control group; ^*p* < 0.05, ^^^*p* < 0.001 vs the CCCP group.

The ATP level was decreased after CCCP induction, but this reduction was mitigated after MA treatment (20, 40, and 80 μM) (Figure 2a). Additionally, the MMP levels decreased after CCCP addition, but this effect was reversed after MA treatment (Figure 2b). Taken together, MA ameliorated mitochondrial function in HGSMCs.

### 3.3 MA restrained autophagy in HGSMCs

**Figure 3:**
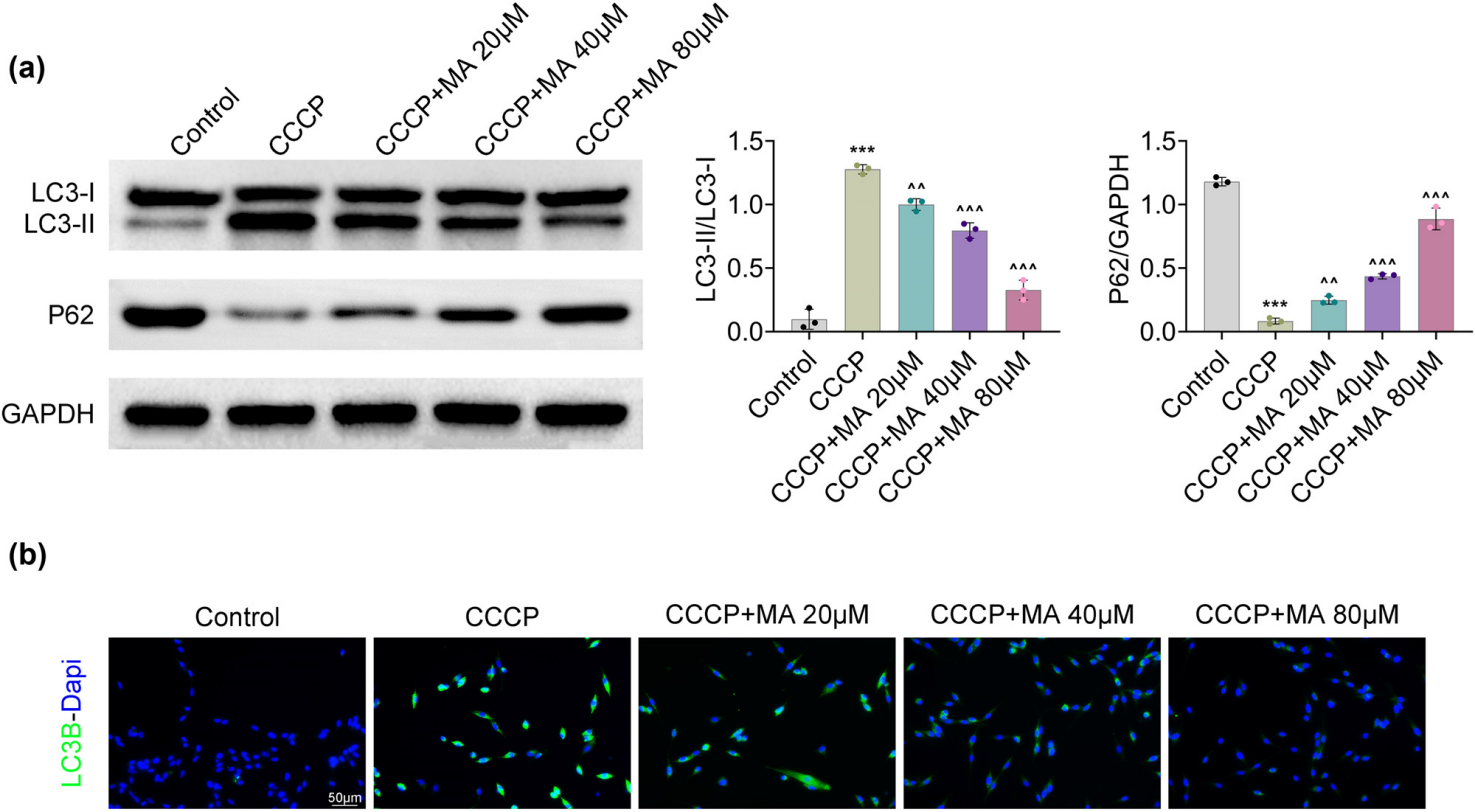
MA restrained autophagy in HGSMCs. Groups were separated into the control, CCCP, CCCP+MA 20 μM, CCCP+MA 40 μM, and CCCP+MA 80 μM group. (a) The protein expressions of LC3-I, LC3-II, and P62 were tested through western blot. (b) The LC3B fluorescence intensity was verified through the IF assay. ***p < 0.001 vs the control group; ^^*p* < 0.01, ^^^*p* < 0.001 vs the CCCP group.

The protein expression of LC3II/LC3I was increased and that of P62 was attenuated after CCCP stimulation, but these impacts were counteracted after MA addition (Figure 3a). Moreover, the LC3B fluorescence intensity was heightened after CCCP induction, but this increase was neutralized after MA treatment (Figure 3a and b). Overall, MA restrained autophagy in HGSMCs.

### 3.4 MA alleviated oxidative stress in HGSMCs

**Figure 4:**
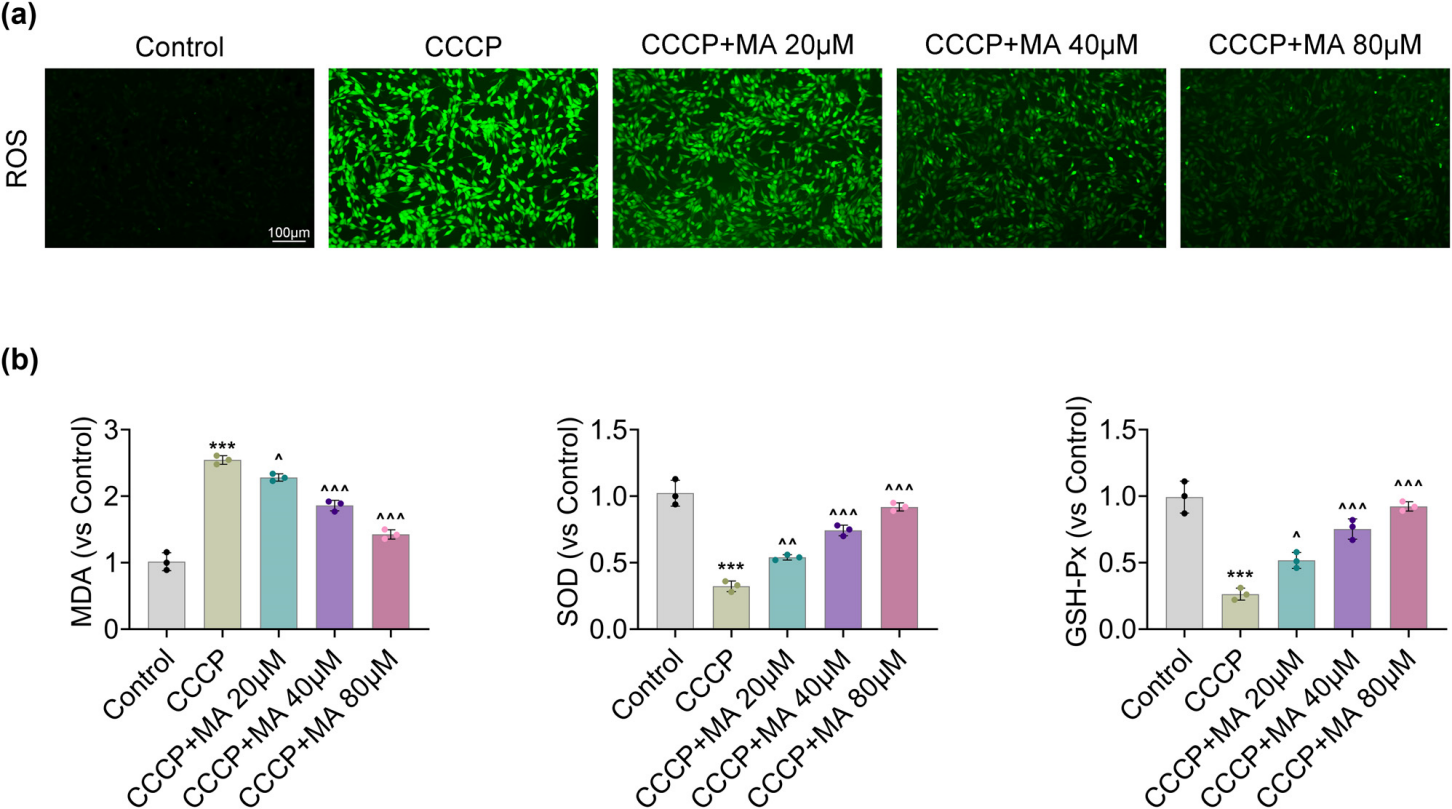
MA alleviated oxidative stress in HGSMCs. Groups were separated into the control, CCCP, CCCP+MA 20 μM, CCCP+MA 40 μM, and CCCP+MA 80 μM group. (a) The ROS fluorescence intensity was measured through the ROS commercial kit. (b) The levels of MDA, SOD, and GSH-Px were examined through the commercial kits. ***p < 0.001 vs the control group; ^*p* < 0.05, ^^*p* < 0.01, ^^^*p* < 0.001 vs the CCCP group.

The ROS fluorescence intensity increased after CCCP treatment, but this effect was reduced after MA addition (Figure 4a). Besides, the levels of malondialdehyde increased, while SOD and GSH-Px levels decreased after CCCP addition, but these effects were rescued after MA treatment (Figure 4b). In summary, MA alleviated oxidative stress in HGSMCs.

### 3.5 MA triggered the AMPK/SIRT1 pathway

**Figure 5:**
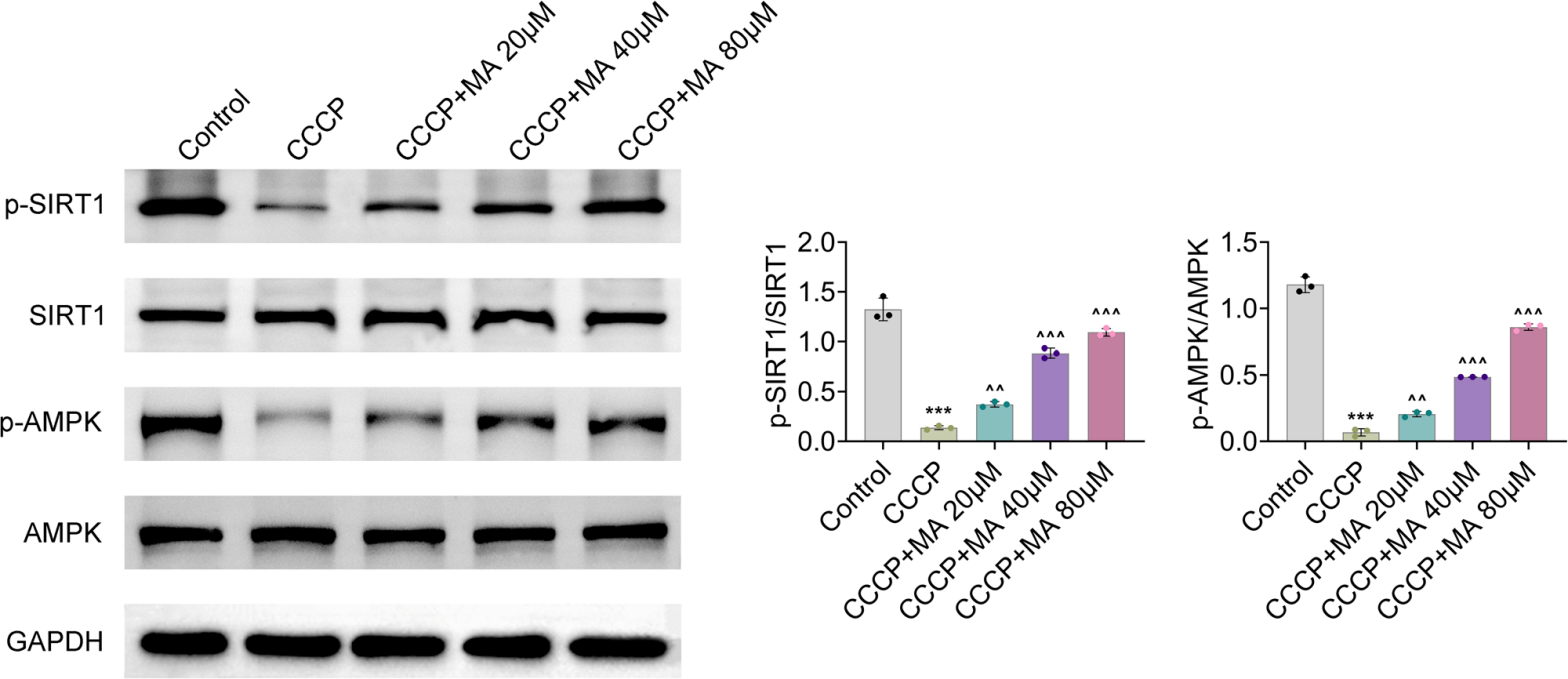
MA triggered the AMPK/SIRT1 pathway. Groups were separated into the control, CCCP, CCCP+MA 20 μM, CCCP+MA 40 μM, and CCCP+MA 80 μM group. The protein expressions of p-SIRT1, SIRT1, p-AMPK, and AMPK were determined through western blot. ***p < 0.001 vs the control group; ^^*p* < 0.01, ^^^*p* < 0.001 vs the CCCP group.

The protein expressions of p-SIRT1/SIRT1 and p-AMPK/AMPK decreased after CCCP stimulation, but these changes were offset after MA addition (Figure 5), indicating that MA activated the AMPK/SIRT1 pathway.

## 4 Discussion

Abundant extracts from Chinese herbs have focused curing FD. For example, magnoloside A can influence brain-gut peptides and gut microbiota to alleviate FD symptoms [14]. In addition, paeoniflorin triggers the release of acetylcholine to ameliorate abnormalities in FD rats [15]. Moreover, hesperidin modulates mitophagy to facilitate gastric motility in FD rats [16]. MA is one pentacyclic triterpene acid and exhibits beneficial effects on the progression of various diseases [7–11]. Nevertheless, the detailed functions and associated pathways of MA in FD progression remain unclear. Similarly, this study demonstrated that MA can accelerate the cell viability of HGSMCs.

Mitochondria are organelles that are highly dynamic, and they can produce ATP and participate in the modulation of oxidative stress and cell apoptosis [17]. Moreover, autophagy plays a pivotal role in maintaining mitochondrial homeostasis [18]. Therefore, maintenance of normal autophagy-mediated mitochondrial functions and oxidative stress is vital for cellular functions [19]. Many researchers have investigated autophagy, mitochondrial functions and oxidative stress in FD progression. For instance, quercetin modulates the PI3K/AKT pathway in FD to improve mitochondrial function and reduce inflammation, thereby relieving H_2_O_2_-triggered oxidative stress damage [20]. Moreover, Chaihu Shugan San affects mitophagy to enhance gastric motility in FD rats [21]. ZhiShiXiaoPi tang retards the mTOR pathway to restrain autophagy-evoked corticosterone to improve FD [22]. In addition, Shen-Ling-Bai-Zhu-San can stimulate the energy metabolism pathways and weaken oxidative stress in FD [23]. Similar to these previous studies, in this project, it was discovered that the strengthened mitochondrial functions stimulated by CCCP induction were restored after MA treatment. Furthermore, autophagy was exacerbated after CCCP addition, but this phenomenon was reversed after MA treatment. The oxidative stress was increased after CCCP treatment, but this effect was alleviated after MA addition.

The AMPK/SIRT1 pathway is a key pathway that is closely associated with autophagy and oxidative stress and has been reported in multiple diseases. For example, pterostilbene influences the AMPK/SIRT1 pathway to restrain oxidative stress and airway inflammation to ameliorate asthma [24]. Additionally, in diabetic kidney disease, metformin affects the AMPK/SIRT1 pathway to relieve oxidative stress and enhance autophagy [25]. Moreover, in dry eye disease, salidroside modulates the AMPK/SIRT1 pathway to stimulate autophagy and mitigate oxidative stress [26]. Importantly, it has been discovered that MA can trigger the AMPK/SIRT1 pathway to improve diabetic nephropathy [27]. However, the regulatory effects of MA on the AMPK/SIRT1 pathway in FD progression remain unclear. In this work, the AMPK/SIRT1 pathway was retarded after CCCP stimulation, but this change was offset after MA addition.

In conclusion, it was uncovered that MA accelerated HGSMCs viability and improved mitochondrial function, inhibited autophagy, alleviated oxidative stress, and stimulated the AMPK/SIRT1 pathway. However, this work also has some limitations, such as a shortage of clinical investigations, human samples, animal experiments, and deep experiments on other phenotypic indicators (autophagy, oxidative stress, mitochondrial function). Therefore, in the future, more experiments will be made.
